# Holding It Together—Patients' Perspectives on Postoperative Recovery When Using an e-Assessed Follow-Up: Qualitative Study

**DOI:** 10.2196/10387

**Published:** 2018-05-25

**Authors:** Karuna Dahlberg, Maria Jaensson, Ulrica Nilsson, Mats Eriksson, Sigrid Odencrants

**Affiliations:** ^1^ School of Health Sciences Faculty of Medicine and Health Örebro University Örebro Sweden

**Keywords:** ambulatory surgical procedures, mobile apps, postoperative period, qualitative research

## Abstract

**Background:**

There is an emerging trend to perform surgeries as day surgery. After a day surgery, most of the recovery period takes place at home, and patients are responsible for their own recovery. It has been suggested that electronic health (eHealth) technologies can support patients in this process. A mobile app has recently been developed to assess and follow up on postoperative recovery after a day surgery.

**Objective:**

The aim of this study was to explore experiences associated with postoperative recovery after a day surgery in patients using a mobile app to assess the quality of their recovery.

**Methods:**

This is a qualitative interview study with an explorative and descriptive design. Participants were recruited from 4 different day surgery units in different parts of Sweden. The study included 18 participants aged >17 years who had undergone day surgery and used the Recovery Assessment by Phone Points, a mobile app for follow-up on postoperative recovery after day surgery. Participants were purposively selected to ensure maximum variation. Semistructured individual interviews were conducted. Data were analyzed using thematic analysis.

**Results:**

A total of two themes and six subthemes emerged from the data: (1) the theme *Give it all you’ve got* with the subthemes *Believing in own capacity*, *Being prepared,* and *Taking action*, where participants described their possibilities of participating and themselves contributing to improving their postoperative recovery; and (2) the theme *The importance of feeling safe and sound* with the subthemes *Feeling safe and reassured*, *Not being acknowledged,* and *Not being left alone*, which describe the importance of support from health care professionals and next of kin.

**Conclusions:**

It is important that patients feel safe, reassured, and acknowledged during their postoperative recovery. They can achieve this themselves with sufficient support and information from the health care organization and their next of kin. Using a mobile app, both for assessment and to enable contact with the day surgery unit during the postoperative recovery period, can improve care and create a feeling of not being alone after surgery. We propose that postoperative recovery starts in the prerecovery phase when patients prepare for their recovery to get the best possible outcome from their surgery.

## Introduction

Day surgery is preferred by many patients because it is thought to be fast and effective and causes minimal interruption to daily life [1]. Patients are admitted and discharged from the day surgery unit on the same day that the surgery is performed or at the latest 24 hours postsurgery [2]. Mortality is low after day surgery [3,4], and benefits such as lower costs, as well as technical advances in surgery and anesthesia, have contributed to the number of day surgeries expanding internationally [5,6]. Day surgery currently accounts for 70% to 75% of all surgeries in Sweden, the United Kingdom, and the United States [2,7,8].

Recovery after surgery starts immediately after completion of surgery and anesthesia and can last up to several months [9]. Recovery can be a time-consuming process that affects the patient’s physical and psychological status [10] and includes regaining their preoperative social, habitual, psychological, and physical functions [9]. During recovery, patients may experience several different surgery and anesthesia-related symptoms, such as nausea, vomiting, pain, dizziness [11], and postoperative cognitive dysfunction [12]. As most of the recovery occurs outside the hospital after day surgery, self-care can be a central part of recovery [13,14].However, it has been reported that patients can feel lonely and unsure about how the recovery is proceeding [15], and some feel that there was a lack of support after discharge [14].

After the patient has been discharged from the day surgery unit, it is common practice to perform a follow-up call on one of the first postoperative days [16,17]. However, the routines differ, and some units do not have a routine for follow-up. Between 10% and 100% (average 56%) of day surgery units in Norway, Sweden, Denmark, Finland, Iceland, the United Kingdom, the Netherlands, and Portugal have been reported to perform postoperative telephone follow-ups [17].

It has been suggested that eHealth technologies can be used to support patients in their recovery after being discharged from the day surgery unit [14]. The use of mobile phones is constantly increasing, and in 2016, there were 7.509 billion mobile cellular subscribers globally [18]. A large increase has also been seen in Sweden, where today, 81% of the population has access to a mobile phone [19]. When used in health care, mobile phones can improve treatment [20] because of the possibility to provide caregiving activities, such as communication, education, and self-care support [21,22], and can be used in disease prevention [20]. Mobile apps have been tested in many different health conditions such as diabetes [23], chronic obstructive pulmonary disease [24], asthma [25], and postoperative recovery [26-29]. The use of a mobile app in the postoperative period has been shown to be feasible [26,27] and to have positive effects on the quality of postoperative recovery [29]. However, it is important to gain a deeper understanding of patients’ perspectives on how the use of a mobile app in the postoperative context may influence their recovery. To our knowledge, patients’ experiences have not previously been described using a qualitative method. Therefore, the aim of this study was to explore the experience of postoperative recovery after day surgery in patients using a mobile app for systematic assessment of the quality of their recovery.

## Methods

### Design

This qualitative study has an explorative and descriptive design with an inductive approach [30]. This study is part of a mixed method study embedded [31] in a multicentre randomized controlled trial (RCT) evaluating cost-effectiveness and other aspects of an e-assessed follow-up using the Recovery Assessment by Phone Points (RAPP) after day surgery, compared with no e-assessed follow-up. The findings have been published in 2 previous papers [28,29]. The RAPP is a mobile app including the Swedish Web version of the Quality of Recovery questionnaire, which measures postoperative recovery [32], as well as asking the question whether the patient wants to be contacted by a nurse, in total consisting of 25 questions. The patients in the intervention group answered the questions on their smartphone daily for 14 days postoperatively. Patients who answered that they wanted to be contacted by a nurse were given a follow-up call within 24 hours (on weekdays only) [33].

### Procedure

The RCT was conducted from October 2015 to July 2016, and the interviews were performed between December 2015 and July 2016. For the RCT, inclusion criteria were adults (aged >17 years) undergoing day surgery, understanding written and spoken Swedish, and having access to a smartphone. Exclusion criteria were visual impairment, known memory impairment or known alcohol abuse, drug abuse, and surgical abortion [34]. Participants were eligible for the interview study if they were allocated to the intervention (RAPP) group and if they had at least once requested contact with a nurse via the RAPP. A total of 91 individuals initiated contact, and of these, 25 were purposively selected to include maximum variation [35] regarding day surgery unit, gender, age, and type of surgery and anesthesia. Because this study was embedded in the RCT, recruitment of participants was done throughout the entire study period and with the aim to conduct the interview 1 month after the surgery. Information about the study was sent out to the selected individuals (n=25) by email 14 to 30 days after surgery. Between 3 and 7 days after the email was sent, they were contacted via phone by the first author (KD) and informed about the study and invited to participate. Furthermore, 18 agreed to participate. Reasons for declining were lack of interest, time constraints, or impaired health. The participants consisted of 8 men and 10 women aged between 21 and 80 years (median age 52 years) who had undergone day surgery under general (n=14) or local/regional anesthesia (n=4). Surgeries performed were general (n=5); orthopedic (n=7); hand (n=5); or ear, nose, and throat surgery (n=1). All participants chose when and where they wanted the interview to be conducted.

### Data Collection

A semistructured interview guide was used, which included open-ended questions [35]. All participants were interviewed face-to-face by the first author (KD), except for 1 participant who was interviewed via Skype. On average, interviews were conducted 36 days after the surgery (range 22-57 days). The interviews were performed in the participants’ home (n=8), at participants’ workplace (n=3), or at the university (n=7) and with only the interviewer and participant present. Initially, a pilot interview was conducted to test the interview guide (Textbox 1). As no changes were made to the guide subsequent to this, the pilot interview was included in the analysis. During the interviews, probing questions were asked such as *You mentioned...Could you tell me more about that?* to gain a deeper understanding. All interviews were audio-recorded and lasted between 30 and 99 min (mean 49 min). After the last interviews, it was found that no new information was obtained, and therefore, the assumption was made that data saturation had been reached. This was confirmed during the data analysis.

### Analysis

The interviews were analyzed using thematic analysis. Inductive semantic analysis included the 6 phases described by Braun and Clarke [36].

#### Phase 1: Familiarizing Yourself With Your Data

The recorded interviews were transcribed verbatim by the first author (KD; n=1) and a professional transcriber (n=17). All transcribed interviews were checked against the recorded interviews by the first author (KD) to ensure accuracy. The transcripts were then read repeatedly by the first and last authors (KD and SO) to familiarize themselves with the data, and notes about the data were made.

#### Phase 2: Generating Initial Codes

Coding was done structurally, interview by interview, and coding data of interest, in view of the aim of the study, were extracted. The coding of the data was performed by the first author (KD), and thereafter, the codes were discussed and refined with the last author (SO).

#### Phase 3: Searching for Themes

The codes were then searched for patterns and grouped into preliminary subthemes and themes. These were discussed with the second author (MJ), who had not taken part in the coding process or the sensemaking of the codes, and the interpretation of the data up to this phase was confirmed.

#### Phase 4: Reviewing Themes

The themes and subthemes were discussed and reviewed to ensure their correspondence with the original data and the aim of the study. This resulted in both dividing and merging of subthemes before the final themes and subthemes were decided.

#### Phase 5: Defining and Naming Themes

The naming of themes and subthemes took place in discussion between all authors to capture the essence of the themes. This included repeated discussions and repeated renaming of the themes and subthemes.

#### Phase 6: Producing the Report

Writing the paper for publication. During the analysis, the researchers moved back and forth between the different phases of the analytical process. All 5 authors participated in phases 4 to 6 [36]. Examples of the analyses are presented in Table 1.

### Ethical Considerations

This study was conducted in line with ethical principles for clinical research and was approved by the regional ethical review board in Uppsala, Sweden (reference number: 2015/262). The participants received both written and verbal information about the study and gave their written consent. They were informed that participating in the study was voluntary and that they would be able to withdraw from the study without this having negative effects on them or their care.

Being interviewed about private experiences during postoperative recovery can be sensitive and can give rise to unpleasant memories. For this reason, there was an opportunity for the participants to contact the researchers by email or mobile phone for further support. None of the participants contacted the researchers after the interviews.

**Interview guide.**

Do you have any previous experience of undergoing surgery?

What type of surgery have you undergone at this time?

Could you describe your experiences of the first days after the surgery?

What were your thoughts when you received information that the surgery would be performed as day surgery?

How was your recovery affected by the fact that it was a day surgery procedure?

If you reflect on your previous surgery and compare with this surgery, can you describe any pros and cons of your postoperative recovery?

How have you used the app during your postoperative recovery?

What do you think about using this type of information technology (IT) solution after surgery?

Can you describe the contact with the nurse, which was initiated via the app?

What were your expectations on this contact?

**Table 1:** Examples from the analysis.

Theme and subtheme		Codes	Data extract
**Give it all you’ve got**			
	Believing in own capacity	Knowing your capacity	*...It’s like when you have experience of having had an operation and coming home; you kind of know what you can manage.*
	Being prepared	Exercising to be more prepared for recovery	*So I went and did a bit of exercise to try to be a bit more...mobile...when... before the operation. Yeah, quicker recovery.*
	Taking action	Self-care for constipation	*...then I was constipated...maybe for the first 3 or 4 days and that was unpleasant. So then I tried to drink a lot and have a lot of fluids and vegetables, and so on; the things I ate were actually very light.*
**The importance of feeling safe and sound**			
	Feeling safe and reassured	Support from RAPP^a^	*It’s fantastic, I can just click in the app and somebody rings me.*
	Not being acknowledged	Frustration because of insufficient information	*...it went very fast, did everything, and maybe that was what was a bit frustrating too—that on top of everything you didn’t know what had been done and what would be done later.*
	Not being left alone	Support from family	*I did get help from...my husband, and my sister brought me food and things...*

^a^RAPP: Recovery Assessment by Phone Points.

## Results

### Overview

A total of 2 themes and 6 subthemes emerged from the data: *Give it all you’ve got* with the subthemes *Believing in own capacity*, *Being prepared,* and *Taking action*; and *The importance of feeling safe and sound* with subthemes *Feeling safe and reassured*, *Not being acknowledged*, and *Not being left alone*.

### Give It All You’ve Got

#### Believing in Own Capacity

As it was decided that the operation would be performed as day surgery, the participants felt chosen and deemed themselves capable of having day surgery. Hence, they wanted to fulfill the expectations from the health care organization that they were capable of undergoing day surgery. This expectation from the health care professionals contributed to a feeling of confidence and strengthened their belief in their own capacity to handle the postoperative recovery. Once the participants felt confident, they felt comfortable and secure and experienced the postoperative period as positive, and they described that being at home after surgery promoted a faster and safer recovery:

...then if it’s just for 1 day—well, then I kind of manage it myself.

Participant 6

Staying positive and encouraging themselves was described as a way to compensate for the feeling of insecurity about handling the recovery process the participants sometimes felt. One thing that facilitated believing in their own capacity was being familiar with the health care system and knowing when and whom to call if support was needed:

...if I want contact with a person I make sure I get it. I usually don’t settle with...if you know what I mean...

Participant 2

Even if they experienced problems during the recovery process, they trusted themselves and believed in their own capacity should they have to undergo day surgery again. Some participants who experienced problems during recovery expressed self-doubt as to whether they should have undergone the surgery at all.

#### Being Prepared

The participants considered barriers and facilitators in their everyday life based either on previous experiences or on assumptions regarding having surgery and recovering at home. When they had negative expectations based on previous negative experiences, it made them more cautious about trusting health care. Many participated actively in the decision making regarding anesthesia and the surgery. They prepared themselves mentally, physically, socially, and practically for the recovery to influence the recovery in the best possible way. These preparations might include strategies such as searching for information, physical training, preparing for rehabilitation, arranging support from next of kin, preparing for school work or housework, having enough food in the house, and preparing food. Being prepared reduced worries and anxiety during the recovery period:

I had prepared things at home in a different way. I had sort of put the pillows up in the bed, and so on...my mother-in-law and mum were more on stand-by in a different way than last time...A bit like, prepared a bit more, maybe in the fridge at home also.

Participant 16

#### Taking Action

The participants were determined to fulfill their desire to recover, get back to normal, and be able to do things that had been impossible before the surgery or during recovery. They described this as allowing the recovery to be important, taking time and letting go of all other things besides the recovery process. The participants described themselves as active participants who took responsibility for their recovery. They handled postoperative symptoms and prevented complications. Some used a relative’s or friend’s pain medications because they perceived their own pain medication as insufficient:

I actually got to borrow my mum’s, she actually had Citodon, I’d used them before and so I did actually take them. Ehm, I took them so I’d sleep a bit better at night, otherwise I’d never have coped.

Participant 1

Some of the participants said that a good recovery was something they themselves were responsible for, not the health care staff. They described feelings of happiness and relief when the recovery proceeded as planned, and they blamed themselves when it did not turn out as expected.

### The Importance of Feeling Safe and Sound

#### Feeling Safe and Reassured

Being treated professionally and as an individual made the participants feel safe. Being treated professionally included being able to get sufficient information, discuss worries, and get reassurance. The participants felt reassured when they received a follow-up call or paid a visit to the health care to confirm that everything was proceeding as expected and get assistance with symptoms and concerns. The option to decide to call the day surgery unit when needed created feelings of safeness and patient participation:

...that they say, “Yes, but this actually looks fine,” it is good to hear that too. It’s almost nicer to hear it...when both the hospital, or yes, the nurse says, “Oh, this looks really good,” that’s quite relieving.

Participant 14

The RAPP was described as a solution that could help deal with negative experiences like those the participants had suffered after previous day surgeries, when it had been difficult to get in contact with health care. The RAPP enabled the participants to report how they felt and further made them reflect on their postoperative recovery. The opportunity to be contacted by a nurse on request was described as improving their postoperative recovery because it created a feeling of safety and of not being left alone after discharge. The participants suggested that the RAPP should be implemented more widely so that all patients could use it in their postoperative period:

I think everybody who is going to have an operation should get the app. You feel better, you can see an improvement...or a worsening.

Participant 13

They related that the nurse reassured them, gave advice, and explained the symptoms or concerns they experienced. The nurse contacted other health care expertise if needed. This alleviated their anxiety and worries during recovery. The support enabled by the RAPP was described as a lifeline because for some, it was the only way to get support from health care in their recovery process:

...was actually my lifeline. “Yes, please contact me.” Because when I felt that something was amiss I just clicked in (the app) and then the nurse rang me up.

Participant 5

#### Not Being Acknowledged

The participants experienced that insufficient information and support as well as lack of acknowledgment from health care created a feeling of being forsaken. This lack of support and information left them by themselves to deal with their worries about symptoms and distress, and this made them feel abandoned by the health care service:

...the pain scale actually goes from 0 to 10. Ten is insufferable...hmm, that doesn’t even cover it. So that...it was really hard. When I rang in the next day, she, the nurse, actually said, “No, you can’t have any [pain medication]!”...But yes, I didn’t get any so every day has been a struggle.

Participant 8

In 1 case, lack of information about the operation had almost spoilt the participant’s chance of recovery and function of the hand because besides not receiving information about what was done during the surgery, the participants had not been told what not to do during the recovery. Insufficient information also made participants feel misled, which could result in more postoperative discomfort such as pain:

Very negative thoughts, a lot of anxiety and kind of anger, and so on, and that’s never good in a healing process—to be surly and sort of unhappy, and so on. It doesn’t actually get better, the pain doesn’t actually feel better if you sort of focus on the wrong things; nothing gets better that way.

Participant 5

The participants experienced that it was a challenge to get in contact with health care. They did not know whom to approach with questions and concerns and were restricted to specific telephone hours for calling. Some described technical issues they had experienced with RAPP. Others had needed to get in contact with a health care professional after the intervention was completed (ie, after 14 days postoperatively). When they did not manage to get in contact, they felt hopeless. A visit to the emergency department seemed the only solution:

We actually have the best care in the world so that it shouldn’t be that difficult to make contact. Right?...maybe I should have managed to get in, I’m actually…well the last thing I thought was kind of, if I don’t get in now then I have to get to the ER [emergency room]...

Participant 7

Some participants felt disappointed in the RAPP. Having to wait 24 hours for a follow-up call seemed too long. Some also felt that they had been unprofessionally treated by the nurse, for instance, when receiving 2 conflicting pieces of advice or getting advice such as “just deal with it.”

This lack of support, concerning information and acknowledgment, created feelings of unsafety and resulted in participants not trusting the health care service, which led to them not wanting to return to hospital or undergo any more surgeries.

#### Not Being Left Alone

During the recovery, the participants experienced distress and symptoms such as dizziness, feeling groggy, nausea, and vomiting, or difficulties with mobility. It was important to have support from a next of kin, and they thought that the recovery would have been difficult, perhaps impossible, without the help and support of next of kin:

...you just want to go home. On the condition of course that you know that you will be taken good care of at home...I am very lucky, I have had it quite easy, I haven’t need to bother about anything...

Participant 4

To feel safe, physically, mentally, and in practical things, the participants needed support. They described their next of kin as someone who provided company and lifted the atmosphere and someone who helped with everyday life, housework, hygiene, and issues related to the surgery. The participants had received advice from their friends and relatives on how to handle issues and symptoms. Some said they were more confident in the support from their next of kin than in the support they got from the health care professionals. Insufficient support from their employer, such as a persistent workload, or not having arranged a substitute, created stress as well as worries and had a negative effect on the recovery. Those participants who were self-employed described it as a challenge to manage demands from their work during recovery because they had no one to support them and had to manage their work on their own.

## Discussion

### Principal Findings

The findings in this study resulted in 2 main themes: *Give it all you’ve got* and *The importance of feeling safe and sound*, both of them describing the experiences of postoperative recovery as well as how an e-assessment can influence postoperative recovery in persons undergoing day surgery. The best conditions for an optimal postoperative recovery may be a balance between *Give it all you’ve got* and *The importance of feeling safe and sound*. The participants described feeling safe and secure as something central and said that these feelings had a positive impact on their recovery. On the basis of the findings in this study, a feeling of safety could be created by efforts from the patients themselves; however, this cannot be done unless the health care services provide patients with sufficient information and support to manage the recovery process by themselves and unless the patients feel supported by health care and/or next of kin. It has previously been described that day surgery patients who report insufficient support from health care tend to have a poorer recovery [37]. It is important for patients to have enough information to manage their recovery [10,15,38-40]. Insufficient information can lead to anxiety and increased pain after surgery [41,42]. Although previous studies [10,15,38-40] have reported the need for sufficient information, this seems still to be a problem. Lack of information can have a negative effect on patients’ recovery because it affects them emotionally and physically as they experience the symptoms as worse. Lack of support and information can result in complications and can even harm the patient when they are not aware of what aspects to be cautious about, as described in this study.

Postoperative recovery is a process starting directly when the surgery and anesthesia have ended [9] and can be divided into 3 phases: early, intermediate, and late recovery. Early and intermediate recovery occurs at the day surgery unit, whereas late recovery occurs at home [43,44].Our suggestion is that the recovery process in fact starts before surgery in a prerecovery phase. Hence, the recovery process starts when the decision is made to undergo surgery and the participant starts to prepare for the surgery and the subsequent recovery process, just like an athlete prepares for a race. The participants in our study took an active part to be prepared mentally, physically, socially, and practically. Our findings are supported by previous studies with day surgery patients and inpatients preparing emotionally and practically for their surgery [15,45]. Being prepared can also be seen as a coping strategy for dealing with the postoperative recovery [46,47]. Therefore, it is important that health care supports patients to start their prerecovery phase, by informing them and discussing how they can best manage their postoperative recovery and deal with and ease postoperative symptoms [42,48,49].

The participants in this study experienced support from health care and described feelings of safety. One reason for this positive result could be the use of RAPP. Previous studies have discussed the importance of support and contact with health care professionals after discharge [10,14,15] and report that effective follow-up can reduce anxiety in patients undergoing day surgery [50]. The results of our main study (in which this study is embedded) show that participants who were allocated to the RAPP group reported significantly better quality of postoperative recovery compared with participants in the control group who did not use the RAPP [29]. This may indicate that the support they experienced from using the RAPP had a positive impact on their postoperative recovery. Day surgery patients need support to manage their symptoms, and they need answers to their questions about the recovery in general, not only on the first postoperative day, as often is the routine. They need the possibility to decide for themselves when in the recovery process they want to have contact with the health care service; they also want an easy way to get this contact if needed. This finding has been confirmed by others [50-52]. Patients have symptoms and recovery-related questions that need attention also during the subsequent 3 to 5 postoperative days [50,52], and it has also been argued by others that patients should have the possibility to get the support they wish for whenever they need it [50].

It is possible that our participants were more positive toward the RAPP because they had experienced the need for initiating contact with a nurse during the recovery. It may also be that the participants in this study experienced more concerns and issues as they had experienced the need to be contacted by a nurse via RAPP. Therefore, the results from this study may not be transferable to all day surgery patients. However, many of the findings in this study can be confirmed by previous studies performed on a day surgery population, as described earlier [14,15,52], as well as positive attitudes toward using a mobile app during the postoperative period [26,27].

Support from health care is important, but the support of next of kin is just as important. On the basis of our clinical experience and the results of this study, as well as earlier research showing that patients may experience anesthesia and surgery-related symptoms and that quality of recovery is poorer on the first postoperative day [53,54], we recommend that patients should always be informed about the importance of not being alone the first night(s) after discharge from surgery. Furthermore, patients need assistance from next of kin during the first postoperative days, or even weeks in some cases, to increase the feeling of safety and help them cope with the recovery [15,47]. In Sweden and many other European countries, it is not mandatory that patients have someone accompanying them for the first 24 hours [17]; however, this is recommended by the International Association of Ambulatory Surgery [55] as well as by researchers [43,56,57]. The patients may underestimate how much help is needed in the postoperative period [51,52] and therefore may not arrange the support they need from next of kin.

Self-care is a central part of handling postoperative recovery after day surgery [15,51]. Our participants described taking great responsibility for their self-care and also believing in their capacity to handle the recovery process.

The ability to cope with and handle the recovery process can be related to a patient’s self-efficacy. Self-efficacy is a person’s beliefs in their own capacity to handle a situation and has 4 different sources: *enactive mastery experience*, *vicarious experience*, *verbal persuasion*, and *psychological and affective states* [58]. The sources of self-efficacy were not used in the analysis in this study as ours was an inductive analysis. However, it seems that our findings reflect the participants’ sources of self-efficacy. Thus, regarding *enactive mastery experience*, the participants used their previous experiences to judge how they would be able to handle the situation and also what actions were needed to influence the recovery in a positive way. Negative experiences resulted in them doubting their capability to undergo surgery again. Regarding *vicarious experience*, participants with no previous experience of undergoing surgery considered their situation and assumed what actions they had to take to enhance their recovery. In *verbal persuasion,* participants felt that health care had faith in them and therefore had the ability to handle the recovery. In *psychological and affective states,* feeling safe and experiencing support from health care staff or next of kin during recovery had a positive impact on recovery. It has been described that self-efficacy has a positive influence on postoperative recovery after hip replacement surgery [59], and it has been suggested that self-efficacy is central for patients undergoing day surgery [60]. Moreover, in 1 report, self-efficacy improved when support after surgery was increased in a telephone follow-up intervention after total knee arthroplasty [61]; in another, a telehealth intervention after coronary artery bypass graft surgery had a similar positive effect [62].

The contact with the nurse via the RAPP function was described as reassuring. It is possible that the nurses knew that the patients were participating in a study and therefore behaved in a special way. One advantage of using RAPP for contact with the nurse is that nurses have the possibility to be prepared and read the patients’ medical records before calling them. Furthermore, we think that it is better for both the nurse and the patient if the nurse performs the follow-up call when they have time to talk to the patient, rather than answering the phone while performing other tasks. Phone call interruptions in primary care have been described as negative for care both by health care staff and patients [63].

Participants were positive toward the use of digital technology during postoperative recovery. One of the inclusion criteria was to have access to a smartphone, which explains why participants were familiar with, and positive toward, the technology. It would be natural to assume that other issues concerning the RAPP would have emerged had we included participants not comfortable with smartphone technology. A barrier to using digital health technology is older age, a lower educational level, a lower income level [64], and low health literacy [65]. However, it has been shown that these sociodemographic factors are becoming less important in the context of using mobile phone–based health solutions [66].

### Methodological Considerations

Only participants who were allocated to the intervention group and had initiated contact with a nurse by using the RAPP function were eligible for inclusion in this study. This inclusion criterion may have affected the findings and also their transferability [67].

The analysis was performed individually by the researchers and was confirmed in the early stages of the analysis process by the second author (MJ) who was not part of the initial analysis. This increases the credibility of the findings [67]. To ensure credibility, all authors discussed the interpretation of the data on several occasions during the analysis process. Quotations from the participants have been included to support the findings. To enhance confirmability [67], the authors’ preunderstanding was taken under consideration, and the authors strove to be open toward the text. Three of the authors (KD, MJ, and UN) had clinical experience, and four authors (KD, MJ, UN, and ME) had research experience on postoperative recovery, day surgery, and research of the RAPP. This may have affected the results as an analysis is always a product of the researcher performing the analysis. On the other hand, two researchers (ME and SO) did not have experience of working clinically with day surgery or postoperative recovery, and SO had never been involved in any research or development involving RAPP. There were a constant reflection and discussion between the authors during the analysis. SO is a senior researcher in qualitative research, and 3 authors had experience of conducting qualitative research (KD, UN, and ME).

### Conclusions

Our findings emphasize the importance of day surgery patients feeling safe, reassured, and acknowledged during their postoperative recovery at home. This can partly be achieved by the patients themselves, when they believe in themselves and prepare for their recovery as well as take actions and responsibility for improving their recovery. However, support, information, acknowledgment, and reassurance from health care staff and next of kin during their recovery are of great importance. Using a mobile app for assessment and to enable contact with the day surgery unit during the postoperative recovery period can improve care and create a feeling of not being left alone. The postoperative recovery starts in the *prerecovery phase* when patients prepare for their recovery and proactively aim for the best possible outcome of the surgery, anesthesia, and postoperative recovery. A balance between patients’ ability to *Give it all you’ve got*, on the one hand, and information and support from health care and next of kin, on the other hand, appears to be the best condition for reaching an optimal postoperative recovery.

## Abbreviations

IT: information technology
RAPP: Recovery Assessment by Phone Points
RCT: randomized controlled trial
